# 5,5′-Bis(diethyl­amino)-2,2′-[2,2-di­methyl­propane-1,3-diylbis­(nitrilo­methylidyne)]diphenol

**DOI:** 10.1107/S1600536810031430

**Published:** 2010-08-11

**Authors:** Reza Kia, Hadi Kargar, Muhammad Nawaz Tahir, Fatemeh Kianoosh

**Affiliations:** a Department of Chemistry, Science and Research Branch, Islamic Azad University, Tehran, Iran; b Department of Chemistry, School of Science, Payame Noor University (PNU), Ardakan, Yazd, Iran; c Department of Physics, University of Sargodha, Punjab, Pakistan

## Abstract

The asymmetric unit of the title compound, C_27_H_40_N_4_O_2_, comprises one mol­ecule of a potentially tetra­dentate Schiff base ligand. The dihedral angle between the two phenyl rings is 67.13 (10)°. Strong intra­molecular O—H⋯N hydrogen bonds generate *S*(6) ring motifs. One terminal methyl among the four diethyl­amino groups is disordered over two positions with the refined site occupancy ratio of 0.660 (7)/0.340 (7).

## Related literature

For standard values of bond lengths, see: Allen *et al.* (1987 ▶). For details of hydrogen-bond motifs, see: Bernstein *et al.* (1995 ▶). For related structures see, Kargar *et al.* (2009 ▶, 2010 ▶).
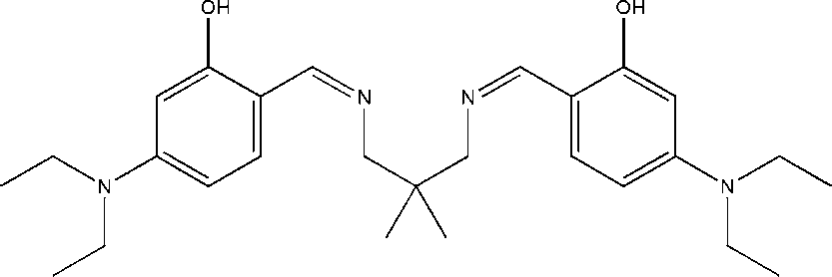


## Experimental

### 

#### Crystal data

C_27_H_40_N_4_O_2_
*M* *_r_* = 452.63
Triclinic, 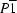

*a* = 10.1143 (5) Å
*b* = 11.4004 (10) Å
*c* = 13.8505 (6) Å
α = 107.572 (3)°
β = 110.771 (2)°
γ = 96.628 (3)°
*V* = 1378.52 (15) Å^3^
*Z* = 2
Mo *K*α radiation
μ = 0.07 mm^−1^
*T* = 296 K
0.27 × 0.21 × 0.11 mm

#### Data collection

Bruker SMART APEXII CCD area-detector diffractometer
Absorption correction: multi-scan (*SADABS*; Bruker, 2005 ▶) *T*_min_ = 0.982, *T*_max_ = 0.992
23024 measured reflections
6522 independent reflections
3856 reflections with *I* > 2σ(*I*)
*R*_int_ = 0.028

#### Refinement

*R*[*F* ^2^ > 2σ(*F* ^2^)] = 0.061
*wR*(*F* ^2^) = 0.199
*S* = 1.06
6522 reflections
304 parameters
2 restraints
H-atom parameters constrained
Δρ_max_ = 0.46 e Å^−3^
Δρ_min_ = −0.29 e Å^−3^

### 

Data collection: *APEX2* (Bruker, 2005 ▶); cell refinement: *APEX2*; data reduction: *SAINT* (Bruker, 2005 ▶); program(s) used to solve structure: *SHELXTL* (Sheldrick, 2008 ▶); program(s) used to refine structure: *SHELXTL*; molecular graphics: *SHELXTL*; software used to prepare material for publication: *SHELXTL*, *PLATON* (Spek, 2009 ▶).

## Supplementary Material

Crystal structure: contains datablocks global, I. DOI: 10.1107/S1600536810031430/jh2191sup1.cif

Structure factors: contains datablocks I. DOI: 10.1107/S1600536810031430/jh2191Isup2.hkl

Additional supplementary materials:  crystallographic information; 3D view; checkCIF report

## Figures and Tables

**Table 1:** Hydrogen-bond geometry (Å, °)

*D*—H⋯*A*	*D*—H	H⋯*A*	*D*⋯*A*	*D*—H⋯*A*
O1—H1⋯N1	0.87	1.76	2.574 (2)	156
O2—H2⋯N2	0.87	1.78	2.587 (2)	153

**Fig. 1.**
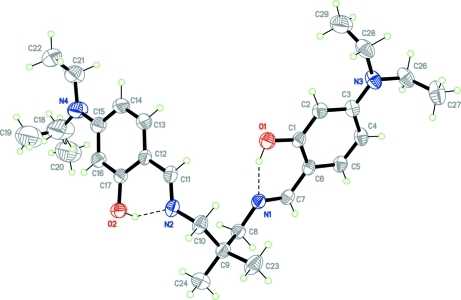
The asymmetric unit of the title compound, showing 30% probability displacement ellipsoids and the atomic numbering. Intramolecular hydrogen bonds are drawn as dashed lines.

## supplementary crystallographic information

### Comment

Schiff base ligands are one of the most prevalent systems in coordination chemistry. As part of a general study of tetradenate Schiff bases (Kargar *et al.,* 2009; Kargar *et al.* 2010), we have determined the crystal structure of the title compound.

The asymmetric unit of the title compound, Fig. 1, comprises a potentially tetradenate Schiff base ligand. The bond lengths (Allen *et al.,* 1987) and angles are within the normal ranges. The dihedral angle between the two phenyl rings is 67.13 (10)°. Strong intramolecular O—H···N hydrogen bonds generate *S(6)* ring motifs (Bernstein *et al.,* 1995). One of the terminal methyl of the diethylamino group was disordered over two positions with the refined site occupancy ratio of 0.660 (7)/0.340 (7).

### Experimental

The title compound was synthesized by adding 4-diethylamino-salicylaldehyde (4 mmol) to a solution of 3,3-dimethylpropylenediamine (2 mmol) in ethanol (20 ml). The mixture was refluxed with stirring for half an hour. The resultant yellow solution was filtered. Yellow single crystals of the title compound suitable for *X*-ray structure determination were recrystallized from ethanol by slow evaporation of the solvents at room temperature over several days.

### Refinement

H atoms of the hydroxy groups were located in a difference Fourier map. They first restrained to 0.85 (1) |%A and then constraied to refine with the parent atoms with U_iso_(H) = 1.5 U_eq_(O), see Table 1. The remaining H atoms were positioned geometrically with C-H = 0.93-0.97 Å and included in a riding model approximation with U_iso_ (H) = 1.2 or 1.5 U_eq_ (C). A rotating group model was used for the methyl groups. One of the terminal methyl of the diethylamino group was disordered over two positions with the refined site occupancy ratio of 0.660 (7)/0.340 (7), and their distances were restrained to be 1.54 (1)Å.

### Crystal data

**Table:**

C_27_H_40_N_4_O_2_	*Z* = 2
*M**_r_* = 452.63	*F*(000) = 492
Triclinic, *P*1	*D*_x_ = 1.090 Mg m^−^^3^
Hall symbol: -P 1	Mo *K*α radiation, λ = 0.71073 Å
*a* = 10.1143 (5) Å	Cell parameters from 2273 reflections
*b* = 11.4004 (10) Å	θ = 2.5–27.5°
*c* = 13.8505 (6) Å	µ = 0.07 mm^−^^1^
α = 107.572 (3)°	*T* = 296 K
β = 110.771 (2)°	Block, yellow
γ = 96.628 (3)°	0.27 × 0.21 × 0.11 mm
*V* = 1378.52 (15) Å^3^	

### Data collection

**Table:**

Bruker SMART APEXII CCD area-detector diffractometer	6522 independent reflections
Radiation source: fine-focus sealed tube	3856 reflections with *I* > 2σ(*I*)
graphite	*R*_int_ = 0.028
φ and ω scans	θ_max_ = 27.9°, θ_min_ = 2.9°
Absorption correction: multi-scan (*SADABS*; Bruker, 2005)	*h* = −13→13
*T*_min_ = 0.982, *T*_max_ = 0.992	*k* = −14→14
23024 measured reflections	*l* = −18→18

### Refinement

**Table:**

Refinement on *F*^2^	Primary atom site location: structure-invariant direct methods
Least-squares matrix: full	Secondary atom site location: difference Fourier map
*R*[*F*^2^ > 2σ(*F*^2^)] = 0.061	Hydrogen site location: inferred from neighbouring sites
*wR*(*F*^2^) = 0.199	H-atom parameters constrained
*S* = 1.06	*w* = 1/[σ^2^(*F*_o_^2^) + (0.0929*P*)^2^ + 0.195*P*] where *P* = (*F*_o_^2^ + 2*F*_c_^2^)/3
6522 reflections	(Δ/σ)_max_ < 0.001
304 parameters	Δρ_max_ = 0.46 e Å^−^^3^
2 restraints	Δρ_min_ = −0.29 e Å^−^^3^

### Special details

**Table:**

Geometry. All esds (except the esd in the dihedral angle between two l.s. planes) are estimated using the full covariance matrix. The cell esds are taken into account individually in the estimation of esds in distances, angles and torsion angles; correlations between esds in cell parameters are only used when they are defined by crystal symmetry. An approximate (isotropic) treatment of cell esds is used for estimating esds involving l.s. planes.
Refinement. Refinement of F^2^ against ALL reflections. The weighted R-factor wR and goodness of fit S are based on F^2^, conventional R-factors R are based on F, with F set to zero for negative F^2^. The threshold expression of F^2^ > 2sigma(F^2^) is used only for calculating R-factors(gt) etc. and is not relevant to the choice of reflections for refinement. R-factors based on F^2^ are statistically about twice as large as those based on F, and R- factors based on ALL data will be even larger.

### Fractional atomic coordinates and isotropic or equivalent isotropic displacement parameters (Å^2^)

**Table:**

	*x*	*y*	*z*	*U*_iso_*/*U*_eq_	Occ. (<1)
O1	0.69136 (14)	0.38335 (13)	0.42554 (10)	0.0739 (4)	
H1	0.7333	0.3242	0.4052	0.111*	
O2	0.72879 (15)	0.03336 (13)	−0.02875 (11)	0.0690 (4)	
H2	0.7166	0.0203	0.0264	0.104*	
N1	0.87493 (16)	0.24580 (14)	0.41808 (12)	0.0556 (4)	
N2	0.66993 (17)	0.06244 (16)	0.14294 (12)	0.0610 (4)	
N3	0.81113 (18)	0.74234 (17)	0.75429 (14)	0.0728 (5)	
N4	0.6665 (3)	0.3442 (2)	−0.19569 (19)	0.1013 (7)	
C1	0.78877 (18)	0.45852 (17)	0.52906 (14)	0.0532 (4)	
C2	0.75271 (19)	0.56052 (17)	0.58747 (15)	0.0573 (5)	
H2A	0.6626	0.5764	0.5544	0.069*	
C3	0.84833 (19)	0.64160 (17)	0.69594 (15)	0.0565 (4)	
C4	0.98449 (19)	0.61456 (19)	0.74217 (15)	0.0624 (5)	
H4A	1.0506	0.6657	0.8140	0.075*	
C5	1.01977 (19)	0.51437 (19)	0.68259 (15)	0.0594 (5)	
H5A	1.1111	0.5001	0.7148	0.071*	
C6	0.92522 (18)	0.43177 (17)	0.57511 (14)	0.0511 (4)	
C7	0.96270 (19)	0.32419 (17)	0.51473 (14)	0.0537 (4)	
H7A	1.0546	0.3111	0.5473	0.064*	
C8	0.9188 (2)	0.13952 (17)	0.36055 (15)	0.0579 (5)	
H8A	1.0083	0.1314	0.4123	0.069*	
H8B	0.9389	0.1564	0.3019	0.069*	
C9	0.8028 (2)	0.01422 (18)	0.31002 (15)	0.0613 (5)	
C10	0.6579 (2)	0.0241 (2)	0.23090 (16)	0.0660 (5)	
H10A	0.5878	−0.0577	0.1978	0.079*	
H10B	0.6206	0.0852	0.2732	0.079*	
C11	0.6259 (2)	0.1596 (2)	0.13056 (15)	0.0608 (5)	
H11A	0.5870	0.2030	0.1781	0.073*	
C12	0.63422 (19)	0.20459 (17)	0.04561 (14)	0.0550 (4)	
C13	0.5885 (3)	0.3123 (2)	0.03497 (18)	0.0744 (6)	
H13A	0.5508	0.3552	0.0836	0.089*	
C14	0.5968 (3)	0.3576 (2)	−0.04428 (19)	0.0803 (6)	
H14A	0.5639	0.4294	−0.0490	0.096*	
C15	0.6546 (2)	0.2968 (2)	−0.11830 (17)	0.0689 (5)	
C16	0.6990 (2)	0.1877 (2)	−0.10934 (16)	0.0640 (5)	
H16A	0.7371	0.1451	−0.1578	0.077*	
C17	0.68801 (18)	0.14135 (17)	−0.03042 (14)	0.0541 (4)	
C18	0.7544 (9)	0.2963 (6)	−0.2568 (6)	0.190 (3)	
H18A	0.8033	0.3652	−0.2691	0.228*	0.660 (7)
H18B	0.8285	0.2640	−0.2131	0.228*	0.660 (7)
H18C	0.7434	0.3441	−0.3044	0.228*	0.340 (7)
H18D	0.6971	0.2116	−0.3052	0.228*	0.340 (7)
C19	0.6677 (11)	0.2019 (8)	−0.3565 (8)	0.203 (3)	0.660 (7)
H19A	0.7275	0.1650	−0.3915	0.305*	0.660 (7)
H19B	0.6027	0.2366	−0.4034	0.305*	0.660 (7)
H19C	0.6118	0.1378	−0.3448	0.305*	0.660 (7)
C20	0.8997 (14)	0.2825 (14)	−0.2338 (13)	0.203 (3)	0.340 (7)
H20A	0.9185	0.2709	−0.2988	0.305*	0.340 (7)
H20B	0.9105	0.2100	−0.2130	0.305*	0.340 (7)
H20C	0.9677	0.3574	−0.1740	0.305*	0.340 (7)
C21	0.6062 (4)	0.4490 (3)	−0.2141 (3)	0.1042 (9)	
H21A	0.6555	0.4870	−0.2501	0.125*	
H21B	0.6263	0.5131	−0.1427	0.125*	
C22	0.4456 (5)	0.4118 (3)	−0.2835 (3)	0.1328 (12)	
H22A	0.4136	0.4853	−0.2927	0.199*	
H22B	0.3954	0.3768	−0.2474	0.199*	
H22C	0.4246	0.3494	−0.3549	0.199*	
C23	0.7744 (3)	−0.0193 (2)	0.4012 (2)	0.0873 (7)	
H23A	0.7392	0.0460	0.4396	0.131*	
H23B	0.8636	−0.0260	0.4527	0.131*	
H23C	0.7027	−0.0989	0.3683	0.131*	
C24	0.8582 (3)	−0.0893 (2)	0.2478 (2)	0.0935 (8)	
H24A	0.7869	−0.1691	0.2154	0.140*	
H24B	0.9482	−0.0958	0.2986	0.140*	
H24C	0.8744	−0.0681	0.1902	0.140*	
C26	0.9098 (2)	0.8278 (2)	0.86660 (19)	0.0831 (7)	
H26A	0.8892	0.9108	0.8794	0.100*	
H26B	1.0095	0.8374	0.8730	0.100*	
C27	0.8970 (3)	0.7822 (3)	0.9535 (2)	0.1080 (9)	
H27A	0.9635	0.8421	1.0255	0.162*	
H27B	0.9203	0.7012	0.9427	0.162*	
H27C	0.7989	0.7736	0.9483	0.162*	
C28	0.6716 (2)	0.7709 (2)	0.7091 (2)	0.0863 (7)	
H28A	0.6475	0.8157	0.7697	0.104*	
H28B	0.5969	0.6919	0.6638	0.104*	
C29	0.6702 (4)	0.8501 (3)	0.6398 (3)	0.1193 (10)	
H29A	0.5755	0.8663	0.6120	0.179*	
H29B	0.6918	0.8055	0.5786	0.179*	
H29C	0.7425	0.9293	0.6846	0.179*	

### Atomic displacement parameters (Å^2^)

**Table:**

	*U*^11^	*U*^22^	*U*^33^	*U*^12^	*U*^13^	*U*^23^
O1	0.0589 (8)	0.0722 (9)	0.0559 (8)	0.0181 (7)	−0.0020 (6)	0.0077 (6)
O2	0.0818 (9)	0.0697 (9)	0.0671 (8)	0.0309 (7)	0.0355 (7)	0.0305 (7)
N1	0.0539 (9)	0.0590 (9)	0.0504 (8)	0.0137 (7)	0.0166 (7)	0.0216 (7)
N2	0.0572 (9)	0.0689 (10)	0.0470 (8)	0.0060 (8)	0.0122 (7)	0.0229 (7)
N3	0.0550 (10)	0.0763 (11)	0.0671 (10)	0.0085 (8)	0.0244 (8)	0.0036 (9)
N4	0.150 (2)	0.1107 (16)	0.1098 (16)	0.0657 (15)	0.0888 (16)	0.0745 (14)
C1	0.0468 (9)	0.0564 (10)	0.0466 (9)	0.0044 (8)	0.0103 (7)	0.0197 (8)
C2	0.0426 (9)	0.0620 (11)	0.0577 (10)	0.0087 (8)	0.0132 (8)	0.0196 (8)
C3	0.0480 (10)	0.0590 (11)	0.0554 (10)	0.0020 (8)	0.0223 (8)	0.0146 (8)
C4	0.0464 (10)	0.0718 (12)	0.0486 (9)	−0.0009 (9)	0.0126 (8)	0.0092 (9)
C5	0.0428 (9)	0.0739 (12)	0.0526 (10)	0.0097 (8)	0.0132 (8)	0.0207 (9)
C6	0.0440 (9)	0.0593 (10)	0.0466 (9)	0.0066 (7)	0.0150 (7)	0.0216 (8)
C7	0.0459 (9)	0.0643 (11)	0.0511 (9)	0.0121 (8)	0.0161 (8)	0.0268 (8)
C8	0.0566 (10)	0.0631 (11)	0.0533 (10)	0.0170 (9)	0.0201 (8)	0.0229 (8)
C9	0.0695 (12)	0.0595 (11)	0.0535 (10)	0.0125 (9)	0.0209 (9)	0.0257 (8)
C10	0.0592 (11)	0.0738 (13)	0.0554 (10)	−0.0011 (9)	0.0149 (9)	0.0279 (9)
C11	0.0534 (10)	0.0763 (13)	0.0468 (9)	0.0143 (9)	0.0178 (8)	0.0189 (9)
C12	0.0523 (10)	0.0624 (11)	0.0445 (9)	0.0132 (8)	0.0151 (8)	0.0181 (8)
C13	0.0930 (15)	0.0820 (14)	0.0638 (12)	0.0393 (12)	0.0418 (11)	0.0297 (11)
C14	0.1088 (18)	0.0752 (14)	0.0775 (14)	0.0426 (13)	0.0456 (13)	0.0395 (12)
C15	0.0793 (14)	0.0735 (13)	0.0661 (12)	0.0218 (11)	0.0358 (11)	0.0339 (10)
C16	0.0673 (12)	0.0755 (13)	0.0611 (11)	0.0245 (10)	0.0345 (10)	0.0285 (10)
C17	0.0482 (9)	0.0573 (10)	0.0502 (9)	0.0119 (8)	0.0149 (8)	0.0182 (8)
C18	0.342 (8)	0.187 (5)	0.202 (5)	0.147 (6)	0.200 (6)	0.153 (5)
C19	0.307 (11)	0.171 (6)	0.208 (8)	0.116 (7)	0.151 (8)	0.096 (6)
C20	0.307 (11)	0.171 (6)	0.208 (8)	0.116 (7)	0.151 (8)	0.096 (6)
C21	0.159 (3)	0.0858 (17)	0.111 (2)	0.0403 (18)	0.078 (2)	0.0623 (16)
C22	0.183 (4)	0.119 (2)	0.104 (2)	0.071 (3)	0.044 (2)	0.0579 (19)
C23	0.0974 (17)	0.0918 (16)	0.0780 (14)	0.0116 (13)	0.0280 (13)	0.0526 (13)
C24	0.115 (2)	0.0673 (14)	0.0896 (17)	0.0288 (14)	0.0346 (15)	0.0240 (12)
C26	0.0659 (13)	0.0758 (14)	0.0754 (14)	0.0015 (10)	0.0266 (11)	−0.0061 (11)
C27	0.101 (2)	0.125 (2)	0.0709 (15)	0.0122 (16)	0.0349 (15)	0.0063 (15)
C28	0.0636 (14)	0.0899 (16)	0.0876 (16)	0.0203 (12)	0.0333 (12)	0.0062 (13)
C29	0.112 (2)	0.110 (2)	0.126 (2)	0.0521 (19)	0.039 (2)	0.034 (2)

### Geometric parameters (Å, °)

**Table:**

O1—C1	1.350 (2)	C14—H14A	0.9300
O1—H1	0.8657	C15—C16	1.396 (3)
O2—C17	1.347 (2)	C16—C17	1.381 (3)
O2—H2	0.8670	C16—H16A	0.9300
N1—C7	1.277 (2)	C18—C19	1.363 (7)
N1—C8	1.446 (2)	C18—C20	1.427 (9)
N2—C11	1.277 (2)	C18—H18A	0.9700
N2—C10	1.451 (2)	C18—H18B	0.9700
N3—C3	1.367 (2)	C18—H18C	0.9600
N3—C28	1.447 (3)	C18—H18D	0.9600
N3—C26	1.458 (3)	C19—H19A	0.9600
N4—C15	1.371 (3)	C19—H19B	0.9600
N4—C21	1.452 (3)	C19—H19C	0.9600
N4—C18	1.474 (5)	C20—H20A	0.9600
C1—C2	1.371 (3)	C20—H20B	0.9600
C1—C6	1.412 (2)	C20—H20C	0.9600
C2—C3	1.404 (2)	C21—C22	1.493 (5)
C2—H2A	0.9300	C21—H21A	0.9700
C3—C4	1.412 (3)	C21—H21B	0.9700
C4—C5	1.359 (3)	C22—H22A	0.9600
C4—H4A	0.9300	C22—H22B	0.9600
C5—C6	1.398 (2)	C22—H22C	0.9600
C5—H5A	0.9300	C23—H23A	0.9600
C6—C7	1.435 (3)	C23—H23B	0.9600
C7—H7A	0.9300	C23—H23C	0.9600
C8—C9	1.527 (3)	C24—H24A	0.9600
C8—H8A	0.9700	C24—H24B	0.9600
C8—H8B	0.9700	C24—H24C	0.9600
C9—C24	1.527 (3)	C26—C27	1.486 (4)
C9—C10	1.528 (3)	C26—H26A	0.9700
C9—C23	1.531 (3)	C26—H26B	0.9700
C10—H10A	0.9700	C27—H27A	0.9600
C10—H10B	0.9700	C27—H27B	0.9600
C11—C12	1.442 (3)	C27—H27C	0.9600
C11—H11A	0.9300	C28—C29	1.501 (4)
C12—C13	1.391 (3)	C28—H28A	0.9700
C12—C17	1.401 (3)	C28—H28B	0.9700
C13—C14	1.370 (3)	C29—H29A	0.9600
C13—H13A	0.9300	C29—H29B	0.9600
C14—C15	1.400 (3)	C29—H29C	0.9600
			
C1—O1—H1	103.9	C19—C18—H18B	109.6
C17—O2—H2	105.6	N4—C18—H18B	109.6
C7—N1—C8	120.34 (15)	H18A—C18—H18B	108.1
C11—N2—C10	118.74 (18)	C19—C18—H18C	80.5
C3—N3—C28	122.36 (17)	C20—C18—H18C	101.9
C3—N3—C26	121.98 (18)	N4—C18—H18C	102.4
C28—N3—C26	115.65 (18)	H18B—C18—H18C	139.6
C15—N4—C21	122.6 (2)	C20—C18—H18D	102.0
C15—N4—C18	119.9 (2)	N4—C18—H18D	103.1
C21—N4—C18	117.2 (2)	H18A—C18—H18D	132.7
O1—C1—C2	118.79 (16)	H18B—C18—H18D	91.3
O1—C1—C6	120.01 (16)	H18C—C18—H18D	105.0
C2—C1—C6	121.20 (15)	H18C—C19—H18D	77.3
C1—C2—C3	121.66 (17)	C18—C19—H19A	109.5
C1—C2—H2A	119.2	H18C—C19—H19A	103.3
C3—C2—H2A	119.2	H18D—C19—H19A	109.1
N3—C3—C2	121.43 (17)	C18—C19—H19B	109.5
N3—C3—C4	121.44 (16)	H18C—C19—H19B	76.9
C2—C3—C4	117.14 (17)	H18D—C19—H19B	137.4
C5—C4—C3	120.56 (16)	C18—C19—H19C	109.5
C5—C4—H4A	119.7	H18C—C19—H19C	141.4
C3—C4—H4A	119.7	H18D—C19—H19C	73.2
C4—C5—C6	123.06 (17)	C18—C20—H20A	109.5
C4—C5—H5A	118.5	C18—C20—H20B	109.5
C6—C5—H5A	118.5	H20A—C20—H20B	109.5
C5—C6—C1	116.37 (16)	C18—C20—H20C	109.5
C5—C6—C7	122.25 (16)	H20A—C20—H20C	109.5
C1—C6—C7	121.37 (15)	H20B—C20—H20C	109.5
N1—C7—C6	122.62 (16)	N4—C21—C22	114.0 (3)
N1—C7—H7A	118.7	N4—C21—H21A	108.7
C6—C7—H7A	118.7	C22—C21—H21A	108.7
N1—C8—C9	112.83 (15)	N4—C21—H21B	108.7
N1—C8—H8A	109.0	C22—C21—H21B	108.7
C9—C8—H8A	109.0	H21A—C21—H21B	107.6
N1—C8—H8B	109.0	C21—C22—H22A	109.5
C9—C8—H8B	109.0	C21—C22—H22B	109.5
H8A—C8—H8B	107.8	H22A—C22—H22B	109.5
C24—C9—C8	108.09 (17)	C21—C22—H22C	109.5
C24—C9—C10	110.27 (17)	H22A—C22—H22C	109.5
C8—C9—C10	110.82 (16)	H22B—C22—H22C	109.5
C24—C9—C23	110.05 (18)	C9—C23—H23A	109.5
C8—C9—C23	110.45 (16)	C9—C23—H23B	109.5
C10—C9—C23	107.16 (17)	H23A—C23—H23B	109.5
N2—C10—C9	113.33 (16)	C9—C23—H23C	109.5
N2—C10—H10A	108.9	H23A—C23—H23C	109.5
C9—C10—H10A	108.9	H23B—C23—H23C	109.5
N2—C10—H10B	108.9	C9—C24—H24A	109.5
C9—C10—H10B	108.9	C9—C24—H24B	109.5
H10A—C10—H10B	107.7	H24A—C24—H24B	109.5
N2—C11—C12	122.42 (18)	C9—C24—H24C	109.5
N2—C11—H11A	118.8	H24A—C24—H24C	109.5
C12—C11—H11A	118.8	H24B—C24—H24C	109.5
C13—C12—C17	116.95 (17)	N3—C26—C27	113.0 (2)
C13—C12—C11	121.55 (18)	N3—C26—H26A	109.0
C17—C12—C11	121.49 (17)	C27—C26—H26A	109.0
C14—C13—C12	122.6 (2)	N3—C26—H26B	109.0
C14—C13—H13A	118.7	C27—C26—H26B	109.0
C12—C13—H13A	118.7	H26A—C26—H26B	107.8
C13—C14—C15	120.6 (2)	C26—C27—H27A	109.5
C13—C14—H14A	119.7	C26—C27—H27B	109.5
C15—C14—H14A	119.7	H27A—C27—H27B	109.5
N4—C15—C16	122.1 (2)	C26—C27—H27C	109.5
N4—C15—C14	120.6 (2)	H27A—C27—H27C	109.5
C16—C15—C14	117.25 (18)	H27B—C27—H27C	109.5
C17—C16—C15	121.80 (18)	N3—C28—C29	112.9 (2)
C17—C16—H16A	119.1	N3—C28—H28A	109.0
C15—C16—H16A	119.1	C29—C28—H28A	109.0
O2—C17—C16	118.60 (17)	N3—C28—H28B	109.0
O2—C17—C12	120.67 (16)	C29—C28—H28B	109.0
C16—C17—C12	120.73 (17)	H28A—C28—H28B	107.8
C19—C18—C20	106.0 (9)	C28—C29—H29A	109.5
C19—C18—N4	110.4 (8)	C28—C29—H29B	109.5
C20—C18—N4	138.8 (9)	H29A—C29—H29B	109.5
C19—C18—H18A	109.6	C28—C29—H29C	109.5
C20—C18—H18A	74.4	H29A—C29—H29C	109.5
N4—C18—H18A	109.6	H29B—C29—H29C	109.5
			
O1—C1—C2—C3	179.27 (16)	N2—C11—C12—C13	−178.62 (18)
C6—C1—C2—C3	−1.0 (3)	N2—C11—C12—C17	1.7 (3)
C28—N3—C3—C2	0.7 (3)	C17—C12—C13—C14	−1.1 (3)
C26—N3—C3—C2	−179.50 (19)	C11—C12—C13—C14	179.3 (2)
C28—N3—C3—C4	−179.2 (2)	C12—C13—C14—C15	−0.8 (4)
C26—N3—C3—C4	0.5 (3)	C21—N4—C15—C16	173.0 (2)
C1—C2—C3—N3	−179.24 (17)	C18—N4—C15—C16	−12.6 (5)
C1—C2—C3—C4	0.7 (3)	C21—N4—C15—C14	−7.2 (4)
N3—C3—C4—C5	−179.63 (17)	C18—N4—C15—C14	167.3 (4)
C2—C3—C4—C5	0.4 (3)	C13—C14—C15—N4	−178.2 (2)
C3—C4—C5—C6	−1.3 (3)	C13—C14—C15—C16	1.6 (3)
C4—C5—C6—C1	1.0 (3)	N4—C15—C16—C17	179.3 (2)
C4—C5—C6—C7	−178.08 (17)	C14—C15—C16—C17	−0.5 (3)
O1—C1—C6—C5	179.89 (16)	C15—C16—C17—O2	177.97 (18)
C2—C1—C6—C5	0.2 (3)	C15—C16—C17—C12	−1.4 (3)
O1—C1—C6—C7	−1.0 (3)	C13—C12—C17—O2	−177.21 (17)
C2—C1—C6—C7	179.25 (16)	C11—C12—C17—O2	2.4 (3)
C8—N1—C7—C6	179.50 (16)	C13—C12—C17—C16	2.2 (3)
C5—C6—C7—N1	177.49 (16)	C11—C12—C17—C16	−178.20 (16)
C1—C6—C7—N1	−1.5 (3)	C15—N4—C18—C19	95.3 (5)
C7—N1—C8—C9	133.19 (17)	C21—N4—C18—C19	−90.0 (5)
N1—C8—C9—C24	177.66 (16)	C15—N4—C18—C20	−55.5 (13)
N1—C8—C9—C10	56.7 (2)	C21—N4—C18—C20	119.2 (11)
N1—C8—C9—C23	−61.9 (2)	C15—N4—C21—C22	−79.2 (3)
C11—N2—C10—C9	−124.9 (2)	C18—N4—C21—C22	106.2 (5)
C24—C9—C10—N2	−63.4 (2)	C3—N3—C26—C27	−86.6 (3)
C8—C9—C10—N2	56.3 (2)	C28—N3—C26—C27	93.2 (3)
C23—C9—C10—N2	176.84 (18)	C3—N3—C28—C29	−85.3 (3)
C10—N2—C11—C12	179.79 (15)	C26—N3—C28—C29	94.9 (3)

### Hydrogen-bond geometry (Å, °)

**Table:**

*D*—H···*A*	*D*—H	H···*A*	*D*···*A*	*D*—H···*A*
O1—H1···N1	0.87	1.76	2.574 (2)	156.
O2—H2···N2	0.87	1.78	2.587 (2)	153.
